# Network biology to artificial intelligence: building the next generation of predictive drug discovery

**DOI:** 10.3389/fimmu.2026.1954008

**Published:** 2026-09-09

**Authors:** Kumar Selvarajoo

**Affiliations:** Key Laboratory of Preclinical Research of Antitumor Drugs, Taizhou Institute of Zhejiang University, Taizhou, Zhejiang, China

**Keywords:** artificial intelligence, digital twins, drug discovery, network biology, precision medicine

## Abstract

Drug discovery remains constrained by high attrition rates, prolonged timelines, and limited ability to translate molecular insights into effective therapies. Although reductionist approaches targeting individual molecules have delivered transformative medicines, successful patient-centric treatment remains a major challenge. Furthermore, increasing evidence demonstrates that therapeutic outcomes emerge from complex, dynamic, and context-dependent biological networks. Systems biology has provided a framework to understand these interactions, yet its predictive capability has been limited by biological complexity, incomplete mechanistic knowledge, and the predominance of associative omics data. Recent advances in artificial intelligence (AI), including machine learning, deep learning, and multimodal foundation models, now offer unprecedented opportunities to integrate increasingly large and heterogeneous biomedical datasets and uncover complex biological relationships. The convergence of AI with systems biology may provide a framework for developing mechanistically informed and testable predictive models that extend beyond target identification toward understanding disease mechanisms, therapeutic responses, and treatment failures. However, mechanistic integration should not be assumed to improve prediction universally. Its value must be evaluated against appropriately matched data-driven models under defined biological conditions. Ultimately, AI-driven systems biology approaches could contribute to the development of dynamic, patient-specific computational representations of biological systems, potentially accelerating precision medicine and transforming drug discovery from empirical experimentation toward predictive, mechanism-guided therapeutic design.

## Introduction

Drug discovery remains one of the most challenging and resource-intensive endeavors in biomedical science. Despite extraordinary advances in molecular biology, omics, structural biology, and pharmaceutical sciences, the journey from target identification to an approved therapeutic typically spans approximately 10–15 years and may require investments on the order of US$1–2 billion or more, depending on the accounting framework and development assumptions (1–4). Yet, the majority of drug candidates fail during clinical development due to inadequate efficacy, unexpected toxicity, or inability to translate findings from experimental models into heterogeneous human populations. Typically, only around 10% of therapeutic candidates entering clinical development ultimately achieve regulatory approval, although this varies depending on the therapeutic area, modality, and stage of development (5). These persistent failures highlight a fundamental limitation of current drug discovery paradigms: biological systems cannot be fully understood or manipulated by examining individual molecular components in isolation (6).

Historically, pharmaceutical discovery has been dominated by a reductionist strategy in which disease-associated alterations in individual genes, proteins, or metabolites are identified and targeted therapeutically. This approach has generated remarkable successes, including targeted kinase inhibitors, hormone therapies, monoclonal antibodies, and molecularly directed treatments. However, the underlying assumption that modulating a single molecular entity will produce a predictable therapeutic outcome is increasingly challenged by the complexity of biological systems (6, 7). Living organisms are organized as highly interconnected networks of molecular interactions, feedback mechanisms, adaptive and emergent responses, where perturbation of one component can propagate through multiple biological layers (8).

In biological networks, molecular components do not contribute equally to system behavior. Certain regulators function as network hubs or bottlenecks, exerting disproportionate influence over cellular states, whereas others represent peripheral nodes with limited impact (9, 10). Consequently, therapeutic intervention against a single target may generate unexpected consequences by disrupting network stability, activating compensatory pathways, or altering physiological processes beyond the intended disease mechanism. This network-level complexity represents a major contributor to drug failure, particularly when preclinical models fail to capture the adaptive behavior of human biological systems (11).

Two historical examples illustrate the limitations of target-centric drug discovery. The cholesterol ester transfer protein (CETP) inhibitor Torcetrapib was developed based on the compelling hypothesis that increasing high-density lipoprotein (HDL) cholesterol would reduce cardiovascular risk (12). Although torcetrapib successfully elevated HDL levels and reduced low-density lipoprotein (LDL) cholesterol, the associated clinical trial was terminated because of increased mortality and cardiovascular events. Subsequent investigations demonstrated that Torcetrapib increased blood pressure and aldosterone through mechanisms that were independent of CETP inhibition, implicating off-target pharmacology rather than an adverse consequence of CETP inhibition itself (13, 14).

Similarly, Rofecoxib was designed as a selective cyclooxygenase-2 (COX-2) inhibitor to reduce inflammation while avoiding gastrointestinal toxicity associated with conventional non-selective anti-inflammatory drugs (15, 16). Although successful clinically, prolonged use revealed increased risks of cardiovascular events, resulting in withdrawal from the market. The mechanism was subsequently linked to disruption of the balance between prostacyclin and thromboxane pathways, illustrating how perturbing one component of a physiological network can produce unintended consequences (17).

These two examples, therefore, illustrate distinct failure mechanisms. Torcetrapib highlights the difficulty of predicting clinically important off-target pharmacology, whereas Rofecoxib illustrates how modulation of an intended molecular target can nevertheless perturb an interconnected physiological network. The distinction is important because a systems-level approach must account not only for on-target pathway effects but also for interactions between the intended pharmacological mechanism and broader biological networks.

These examples also emphasize a fundamental principle: therapeutic response is ultimately a property of biological systems rather than individual molecular targets alone. This realization stimulated the emergence of systems biology and network pharmacology, which aim to describe diseases as dynamic interactions among genes, proteins, metabolites, cells, and physiological processes. Rather than asking *“Which molecule is abnormal or abberated?”*, systems biology asks *“How does the organization of biological networks generate disease states, and how can these networks be therapeutically rewired?”*

## Systems biology: a powerful concept yet awaiting predictive capability

Systems biology represented a paradigm shift from cataloguing molecular abnormalities toward understanding biological organization. Through mathematical modelling, network reconstruction, and multi-omics integration, systems biology provides a framework for describing complex biological processes across multiple scales. Despite substantial advances in quantitative modelling (18, 19), however, relatively few examples exist in which a computational systems model has directly and prospectively determined a therapeutic intervention that subsequently translated into clinical use. This does not imply that systems biology has lacked translational impact: systems-level approaches have contributed to target prioritization, biomarker discovery, pathway analysis, metabolic engineering, drug-response modelling, and rational therapeutic combinations. Rather, the remaining challenge is to establish reproducible examples in which mechanistic models generate prospective predictions that can be experimentally and clinically validated.

The primary limitation has been that biological complexity has exceeded our ability to quantitatively model it. Biological networks are dynamic, context-dependent, and highly nonlinear. The same signaling pathway may produce different outcomes depending on cellular lineage, developmental state, metabolic environment, immune context, or disease stage (20, 21). Current models frequently lack sufficient quantitative parameters describing reaction kinetics, molecular concentrations, spatial organization, and temporal regulation (22). As a result, many models remain descriptive rather than predictive.

Furthermore, although modern omics technologies have generated unprecedented molecular datasets, most measurements represent associations rather than causal mechanisms (23, 24). Differentially expressed genes, altered metabolites, or dysregulated pathways may represent drivers of disease, downstream consequences, or compensatory responses. Without causal information, biological networks reconstructed from observational data may fail to identify the true therapeutic intervention points.

This distinction between association and causation is particularly important in AI-driven drug discovery (25, 26). A model may accurately predict treatment response without capturing the biological mechanisms underlying that response. Conversely, a mechanistically plausible model may perform poorly if its underlying assumptions are incomplete or incorrect. The challenge is not simply to integrate AI with biological knowledge, but to determine when mechanistic information improves prediction, how such information should be encoded, and how models should respond when mechanistic assumptions conflict with experimental observations. Importantly, the benefits of mechanistic integration are likely to depend on the quality and completeness of the underlying mechanistic prior: well-supported constraints may enhance biological plausibility and interpretability, whereas incomplete or misspecified mechanisms may introduce systematic bias or unnecessarily restrict model flexibility (27, 28).

Recent primary studies provide emerging empirical examples of this hybrid approach. Physics-informed and gray-box frameworks have combined machine learning with mechanistic equations and symbolic regression to identify and constrain biological system models (29). Similarly, hybrid machine-learning and mechanistic pharmacokinetic models have been used to predict human drug disposition, demonstrating how mechanistic structure can be incorporated directly into data-driven prediction (30). These studies suggest that mechanistic constraints can be operationalized within AI models, but they also reinforce the need for rigorous benchmarking against appropriately matched purely data-driven alternatives.

Accordingly, mechanistic integration should not be assumed to improve predictive performance *a priori*. Its value should be evaluated using predictive accuracy, calibration, out-of-distribution generalization, robustness to missing data, and performance across independent biological systems and contexts.

## Artificial intelligence: the missing computational engine?

More recently, advances in artificial intelligence (AI), particularly machine learning and deep learning, have begun to transform the landscape of drug discovery by enabling the analysis of biomedical data at reasonable scale and resolution (31–33). Unlike conventional computational approaches that rely primarily on predefined hypotheses or mechanistic assumptions, AI excels at identifying complex, nonlinear relationships from diverse, high-dimensional datasets, including genomic, transcriptomic, proteomic, metabolomic, imaging, electronic health record, and chemical structure data. These capabilities have substantially expanded opportunities for target identification, biomarker discovery, drug repurposing, *de novo* molecular design, toxicity prediction, clinical trial optimization, and patient stratification (34).

The emergence of foundation models provides a further extension of this computational paradigm. Rather than training separate models for individual prediction tasks, foundation models learn reusable representations from very large datasets and can subsequently be adapted to multiple downstream drug-discovery tasks. For example, VideoMol was pretrained on 120 million molecular frames representing approximately 2 million molecules and demonstrated performance across 43 drug-discovery benchmark datasets, including molecular property and target prediction (31). Such studies provide empirical evidence that large-scale self-supervised representation learning can extend AI beyond task-specific prediction toward more generalizable molecular modeling.

Nevertheless, the growing predictive capacity of AI should not be equated with biological understanding (35, 36). A model can achieve high predictive accuracy while relying on correlations that are unstable across diseases, populations, experimental platforms, or clinical settings. This limitation motivates a complementary role for systems biology: rather than replacing data-driven learning, mechanistic modeling can provide biological structure against which AI-derived predictions can be evaluated and experimentally tested.

The convergence of AI with systems biology represents a particularly powerful paradigm for next-generation therapeutic discovery. While AI provides powerful data-driven methods for pattern recognition and prediction, systems biology contributes mechanistic insight by describing the causal interactions among genes, proteins, metabolites, cells, and tissues that collectively govern disease progression and therapeutic response. Together, these complementary approaches enable the construction of predictive, multiscale models that not only identify candidate drug targets but also explain the biological mechanisms underlying their effects.

Recent mechanistically constrained and hybrid approaches demonstrate that this integration can be operationalized rather than remaining purely conceptual, including frameworks that combine neural networks, mechanistic equations, and symbolic regression for biological system identification (29) and hybrid machine-learning/mechanistic models for pharmacokinetic prediction (30). Such mechanistically informed AI models may improve model interpretability, enhance prediction robustness, and facilitate the prioritization of therapeutic strategies with greater biological plausibility.

**Figure 1:**
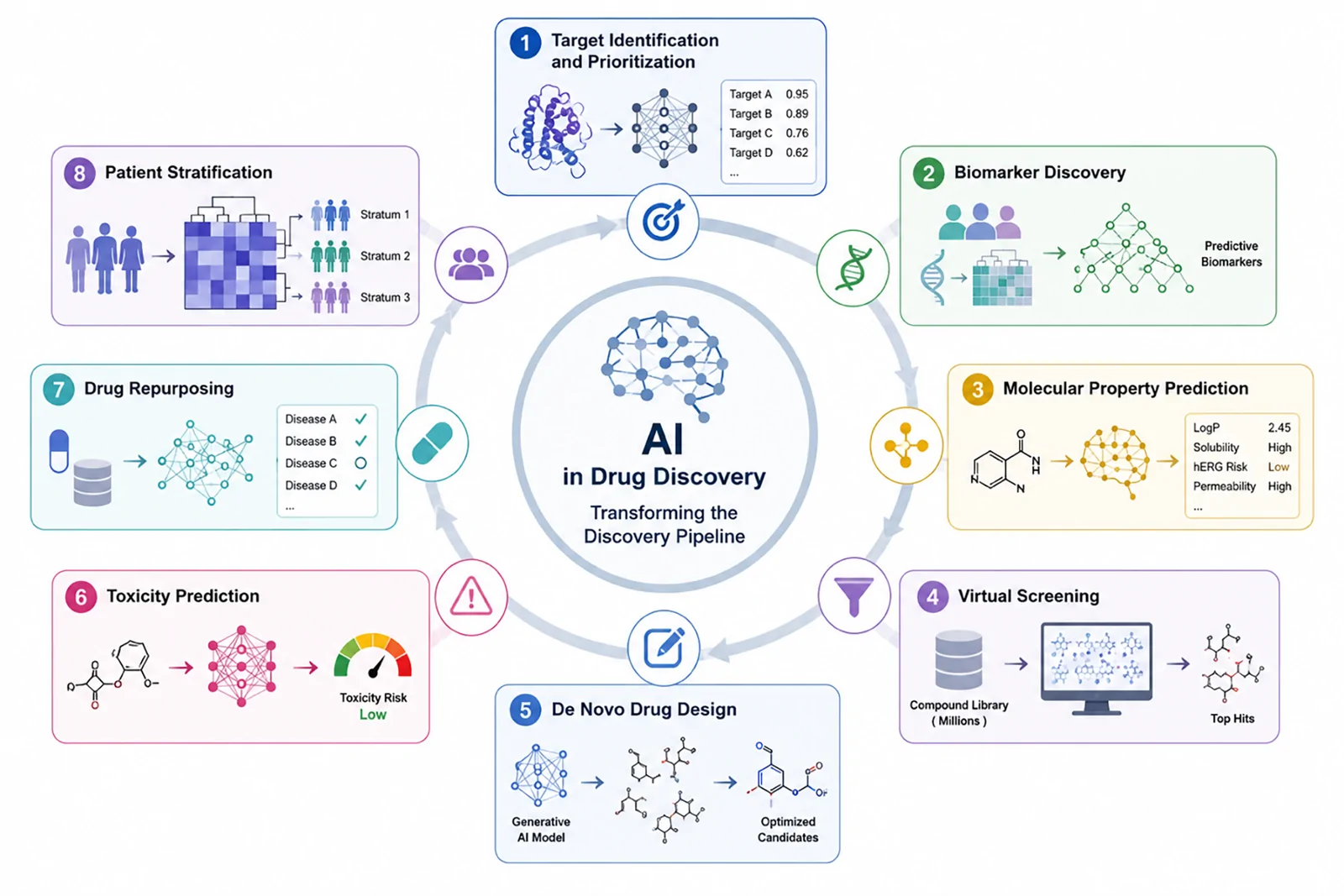
AI in drug discovery. Artificial intelligence is transforming multiple stages of the drug discovery pipeline, including: Step 1, target identification and prioritization; Step 2, biomarker discovery; Step 3, molecular property prediction; Step 4, virtual screening; Step 5, *de novo* drug design; Step 6, toxicity prediction; Step 7, drug repurposing; and Step 8, patient stratification. The extent of experimental and clinical validation differs across these applications.

As increasingly comprehensive multimodal datasets become available, integrating AI with systems biology could shift parts of the drug discovery process from primarily empirical prediction toward models that incorporate biological constraints and explicit hypotheses about mechanism (37, 38). Such models may assist researchers in characterizing disease heterogeneity, anticipating treatment responses, optimizing combination therapies, and stratifying patients according to molecular and cellular characteristics (**Figure 1**).

Importantly, the maturity of AI applications differs substantially across these areas. Some applications, such as molecular property prediction and virtual screening, have extensive computational benchmarking, whereas prospective clinical evidence remains more limited for many other applications. Therefore, claims regarding the impact of AI should be evaluated according to the level of evidence available rather than treating AI-enabled drug discovery as a uniformly mature technology. Therefore, AI-driven drug discovery faces a fundamental limitation: prediction does not necessarily equal understanding. Many AI models achieve high predictive performance while providing limited insight into biological mechanisms. Their reliance on statistical correlations can result in poor generalization when applied to new diseases, populations, or experimental conditions.

## Challenges and future perspective: toward predictive and digital drug discovery

Several challenges remain before systems biology and AI achieve their full potential in drug discovery. First, biological datasets are often heterogeneous, incomplete, and collected using different experimental platforms. Integrating these datasets requires sophisticated computational methods capable of handling missing information and experimental variability.

Second, many AI algorithms function as “black boxes,” producing accurate predictions without explaining their biological reasoning. Explainability is important, but interpretability should also be evaluated against experimentally testable biological mechanisms rather than treated solely as a desirable visualization property (39).

Third, computational predictions require extensive experimental and clinical validation before therapeutic implementation. AI should, thus, be viewed as a decision-support technology rather than a replacement for laboratory research.

A fourth challenge is model uncertainty and biological context. A model trained on one disease, tissue, population, or experimental system may not generalize to another. Future AI–systems biology frameworks will need explicit representations of context, uncertainty, and model applicability.

A fifth challenge concerns model updating. Biological systems are not static, and new experimental observations may invalidate existing mechanistic assumptions. Future systems should support iterative updating in which experimental results modify network structure, model parameters, or uncertainty estimates.

Recent graph-based and retrieval-augmented AI frameworks for clinical decision support illustrate one possible direction for operationalizing structured biological knowledge within AI systems (40, 41). Such approaches provide examples of how graph representations and external knowledge can be quantitatively benchmarked against conventional data-driven approaches. The broader principle is that biological knowledge should become an evaluable component of AI systems rather than an informal layer of interpretation.

Likewise, emerging experimental platforms such as organ-on-chip systems provide opportunities to test network-level predictions in controlled multicellular environments before translation into clinical studies (42, 43). Integrating computational prediction with such intermediate experimental systems could establish a more rigorous feedback loop between in silico models and human-relevant biology.

This progression represents a shift from static molecular target discovery toward dynamic therapeutic systems modelling, in which AI and systems biology are integrated not merely to identify what is associated with disease, but to predict how biological systems will respond to intervention and how those predictions can be experimentally and clinically refined.

The challenges motivate several fundamental research questions that should guide the development and evaluation of AI–systems biology approaches:

Research Question 1: When do mechanistically constrained AI models outperform purely data-driven models?

Mechanistic constraints should not be assumed to improve predictive performance *a priori*. Their value should be evaluated through systematic comparisons with appropriately matched data-driven models using predictive accuracy, calibration, out-of-distribution generalization, robustness to missing data, and performance across independent biological systems as key evaluation criteria.

Research Question 2: How can causal information be distinguished from associative information in multimodal biological datasets?

Addressing this question requires validation beyond statistical association. Evaluation should incorporate intervention-based experiments, perturbation data, causal consistency across independent datasets, and prospective prediction of previously unobserved biological responses. The ultimate test of a proposed causal relationship should be whether manipulating the predicted causal component produces the expected downstream effect.

Research Question 3: How should uncertainty be quantified in AI–systems biology models?

Future models should report not only predictions but also the uncertainty associated with incomplete observations, model misspecification, biological heterogeneity, measurement error, and extrapolation beyond the training distribution. Explicit uncertainty estimation will be particularly important when models are used to prioritize therapeutic interventions or make predictions for biological contexts that are poorly represented in the training data.

Research Question 4: What validation framework is required before a biological digital twin can support therapeutic decisions?

A biological digital twin (see below) should not be considered clinically actionable solely because it reproduces historical observations. We propose a staged validation framework progressing from molecular-level validation and cellular perturbation studies to multicellular and organ-level systems, retrospective patient validation, prospective clinical validation, and ultimately prospective clinical utility studies. Each stage should establish predefined performance criteria before progression to the next level of biological or clinical complexity.

Research Question 5: Can network-level models predict therapeutic response and failure across patients rather than merely classify disease states?

A major test of clinical relevance will be whether network-level models can prospectively predict treatment response, resistance, toxicity, and combination-therapy outcomes in independent patient cohorts. Moving beyond disease classification toward patient-specific prediction will require models that capture both conserved biological mechanisms and clinically relevant sources of interindividual heterogeneity.

## Toward biological digital twins

A longer-term transition for predictive models is towards dynamic biological digital twins creation. For a medical digital twin, it should not be understood simply as a computational replica of a patient. Recent frameworks emphasize an architecture involving the patient, a data connection, a patient-in-silico representation, an interface, and synchronization between the physical and virtual systems (32, 44).

Within drug discovery and precision medicine, such a system could integrate molecular, cellular, experimental, and clinical observations to represent an evolving disease state and simulate candidate interventions. Subsequent experimental or clinical observations could then be used to update the model, creating a closed computational–experimental feedback loop. The digital twin therefore represents not merely a more sophisticated prediction model, but a potential architecture for continuously connecting biological observations, mechanistic hypotheses, intervention simulations, and validation.

**Figure 2:**
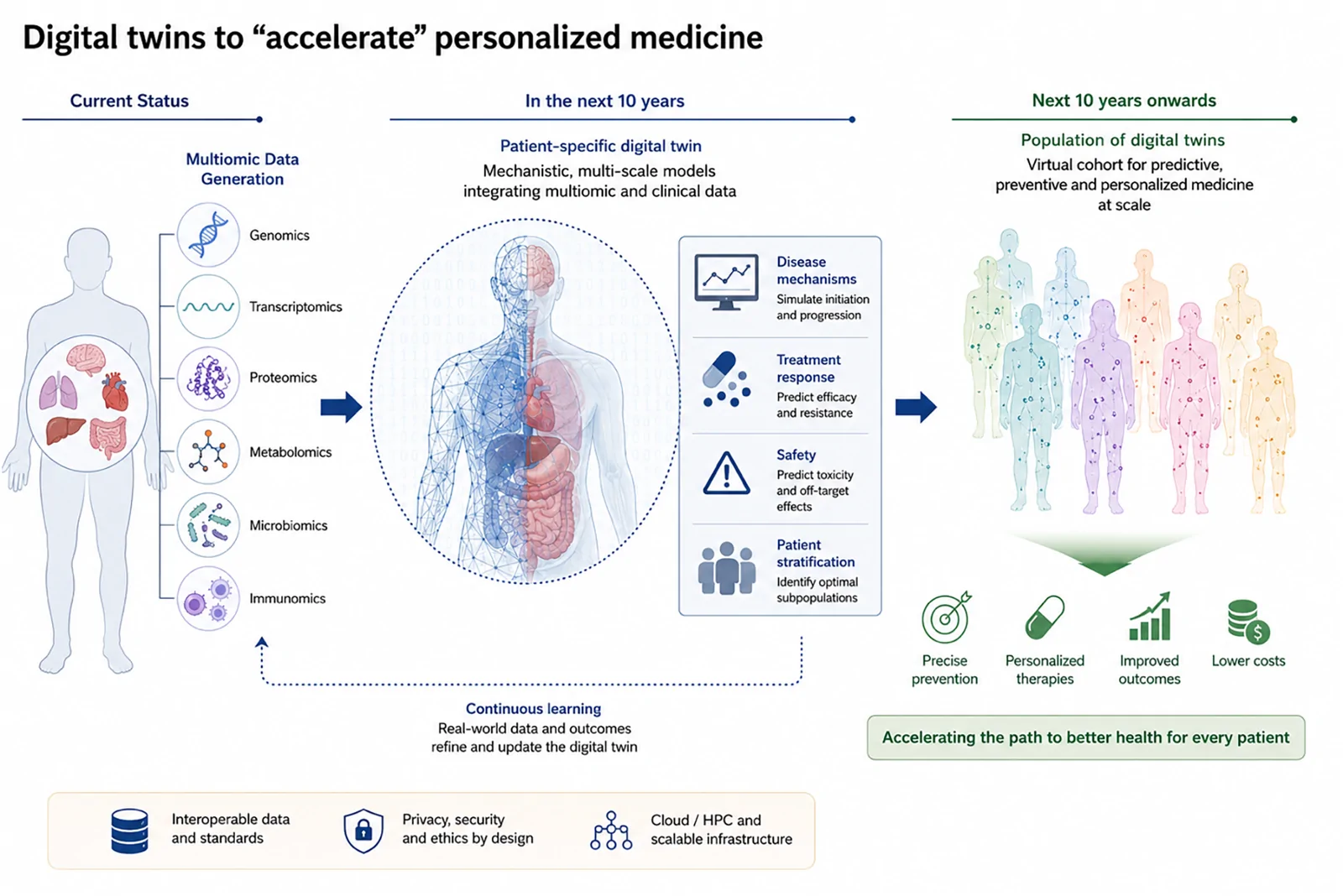
Toward a digital twin approach for personalized medicine. Multi-modal, multi-omics, and multicellular data are integrated with next-generation artificial intelligence and systems biology frameworks to construct patient-specific digital twins, enabling more accurate patient stratification, individualized therapeutic interventions, and continuous refinement of predictive models for precision medicine.

Realizing this vision, however, will require not only advances in AI and systems biology but also rigorous experimental validation, uncertainty quantification, interoperability, and clearly defined standards for demonstrating clinical utility (**Figure 2**).

## From empirical drug discovery to predictive therapeutic design

Recent advances in network pharmacology, molecular simulation, and multi-omics network analysis have increasingly moved drug discovery beyond single-target models toward systems-level representations of disease and therapeutic response. For example, recent works have highlighted the value of integrating network pharmacology with molecular simulation to characterize drug–target interactions and improve mechanistic understanding during drug development (45). Similarly, multi-omics network biomarkers have been used to identify disease-associated molecular modules (46) and support drug repositioning (47). These approaches demonstrate the growing value of network-based representations for target discovery, mechanism characterization, and therapeutic prioritization. However, the emphasis of these approaches remains largely on describing network relationships, identifying candidate targets or biomarkers, and prioritizing existing therapeutic hypotheses.

The perspective developed here differs along several related but important axes. First, rather than treating biological networks primarily as analytical representations, we propose using them as predictive models of perturbation and response (48). Second, rather than viewing AI mainly as a tool for pattern recognition, prediction, or candidate prioritization, we emphasize its integration with mechanistic constraints, causal relationships, and structured biological knowledge (22). Third, we propose a closed-loop framework in which computational predictions are experimentally tested, new observations are incorporated into the model, and the resulting system is iteratively refined. Finally, the framework extends beyond target discovery or drug repositioning toward patient-specific therapeutic prediction, including treatment response, compensatory pathway activation, resistance, toxicity, and treatment adaptation. Thus, the central contribution here is not simply the convergence of AI with systems biology, but the proposition that this convergence should evolve toward dynamic, experimentally validated therapeutic systems modelling.

The goal is not to replace experimental biology or clinical medicine, but to create an intelligent feedback loop between computation and experimentation. AI can generate hypotheses, systems biology can provide mechanistic interpretation, and experimental validation can refine predictive models. Through this iterative process, drug discovery may transition from empirical screening toward rational, predictive, and personalized therapeutic design.

This framework changes the role of computation within drug discovery. Rather than asking only whether a single molecule is associated with a disease phenotype or whether a compound binds a particular target, the objective becomes to predict the system-level consequences of perturbing a biological state. A model should therefore be able to address questions such as: Which target should be perturbed? What network state will result from that perturbation? Which compensatory pathways are likely to be activated? Which patient subpopulation is most likely to respond? Under what biological conditions is resistance likely to emerge? and How should treatment be adapted in response to the predicted or observed response?

Addressing these questions requires models that integrate molecular interactions with cellular states, tissue and disease context, patient heterogeneity, prior treatment history, and temporal changes in biological state. The ultimate objective is therefore to move from static descriptions of molecular relationships toward models capable of predicting how biological systems dynamically change or evolve in response to therapeutic intervention.

## Conclusion

The convergence of systems biology and artificial intelligence represents a potentially important methodological development in predictive drug discovery, but its value should be established through rigorous comparative, perturbational, and prospective evaluation rather than assumed from conceptual complementarity alone.

The central opportunity is to combine the scalability of AI with the mechanistic structure of systems biology to develop models that can learn from multimodal biological data while making explicit, testable predictions about biological perturbations. The critical scientific test is whether such models provide demonstrable gains in prediction, causal inference, generalization, and therapeutic decision-making compared with appropriately matched data-driven alternatives.

Achieving this will require shared benchmarks, causal and perturbational validation, explicit uncertainty quantification, experimental feedback, and prospective clinical evaluation. If these requirements can be met, AI-driven systems biology could provide a foundation for increasingly predictive biological models and, ultimately, patient-specific digital twins. The transition from empirical drug discovery to predictive therapeutic design will therefore depend not on AI or systems biology alone, but on establishing when their integration produces measurable gains in biological understanding, predictive performance, and translational utility.
